# Association between sleep duration and possible sarcopenia in middle-aged and elderly Chinese individuals: evidence from the China health and retirement longitudinal study

**DOI:** 10.1186/s12877-024-05168-x

**Published:** 2024-07-11

**Authors:** Linfeng Chen, Qingyun Li, Xiaoyun Huang, Zhong Li

**Affiliations:** 1 Department of Neurology, The Sixth Affiliated Hospital, Sun Yat-sen University, Guangzhou, 510655 China; 2 Guangdong Medical University, Zhanjiang, 524023 China; 3 Key Laboratory of Human Microbiome and Chronic Diseases (Sun Yat-sen University), Ministry of Education, Guangzhou, 510655 China; 4 Biomedical Innovation Center, The Sixth Affiliated Hospital, Sun Yat-sen University, Guangzhou, 510655 China; 5 Shenzhen Research Institute, Sun Yat-Sen University, Shenzhen, 518000 China; 6 Guangdong Provincial Key Laboratory of Brain Function and Disease, Guangzhou, 510080 China

**Keywords:** Sleep duration, Possible sarcopenia, CHARLS, Longitudinal study

## Abstract

**Background:**

Sarcopenia is a common cause of disability in the aging population, and managing sarcopenia is an important step in building intrinsic capacity and promoting healthy aging. A growing body of evidence suggests that sleep deprivation may be a mediator of the development of sarcopenia. The purpose of this study was to explore the longitudinal association between sleep duration and possible sarcopenia using data from a national sample.

**Methods:**

Two waves of data from the CHARLS database for 2011 and 2015 were used in this study. All possible sarcopenia participants met the Asia Working Group for Sarcopenia 2019 (AWGS 2019) diagnostic criteria. Sleep duration was assessed using a self-report questionnaire, and sleep duration was categorized as short (≤ 6 h), medium (6–8 h), or long (> 8 h) based on previous studies. Longitudinal associations between sleep duration and possible sarcopenia will be calculated by univariate and multifactorial logistic regression analyses and expressed as odds ratios (ORs) and 95% confidence intervals (CIs).

**Results:**

A total of 5654 individuals participated in the follow-up study, with a prevalence of possible sarcopenia of 53.72% (578) in the short sleep duration group, 38.29% (412) in the medium sleep duration group, and 7.99% (86) in the long sleep duration group. According to the crude model of the second-wave follow-up study, short sleep durations were significantly more strongly associated with possible sarcopenia than were medium and long sleep durations (OR: 1.35, 95% CI: 1.17–1.55, *P* = 0.000). The association between short sleep duration and possible sarcopenia was maintained even after adjustment for covariates such as age, gender, residence, education level, BMI, smoking status, alcohol consumption and comorbidities (OR: 1.18, 95% CI: 1.02–1.36, *P* = 0.029). In the subgroup analysis, short sleep duration was associated with low grip strength (OR: 1.20, 95% CI: 1.02–1.41, *P* = 0.031).

**Conclusions:**

Sleep deprivation may be closely associated with the development of possible sarcopenia in middle-aged and elderly people, which provides new insights and ideas for sarcopenia intervention, and further studies are needed to reveal the underlying mechanisms involved.

## Introduction

The global population is aging rapidly due to increasing life expectancy and declining fertility [1]. The rapid aging of the population has led to a significant increase in the total number of people with care needs in the future [2]. Sarcopenia is a common cause of caregiving burden in the aging population [3], and managing sarcopenia is an important step in building the intrinsic capacity to facilitate a disease-centered to function-centered paradigm shift and achieve healthy aging [4, 5].

Sarcopenia is a complex syndrome of aging characterized by low muscle mass, muscle strength or physical performance [6]0.2018 The European Working Group on Sarcopenia updated the definition of sarcopenia and highlighted muscle strength as the main determinant [7]. Muscle strength mostly peaks in young and early adulthood (18–24 years), is maintained during midlife (25–44 years), and declines from midlife (45 + years) [8]. It is estimated that sarcopenia affects 10–16% of older adults worldwide [9]. Changes in muscle structure associated with increased age may be a key driver of a range of adverse health outcomes in older adults, such as falls, fractures, cognitive decline, and death [9, 10]. To identify those at risk of sarcopenia early and provide timely management, the Asian Working Group for Sarcopenia (AWGS) 2019 consensus introduced the concept of “possible sarcopenia” [11]. Loss of muscle mass or poor physical function that can be assessed with low-cost, easily applied techniques for community screening and clinical practice is referred to as possible sarcopenia [11]. This idea was created to enhance patients’ quality of life and assist in more effectively managing the danger of sarcopenia. Therefore, active exploration of the various risk factors contributing to the burden of sarcopenia should be a priority health matter.

The structure and continuity of sleep in middle-aged and older adults change with age [12, 13]. 50% of older adults complain of sleep disturbances [14]. Inadequate or excessive sleep duration is associated with adverse health outcomes in older adults, such as dementia, diabetes, cardiovascular disease, coronary heart disease, obesity, and death [15–17]. Several previous studies have linked short sleep duration and poor sleep quality to multiple dimensions of skeletal muscle damage [18–20]. Recent reports have also shown that imbalances in sleep homeostasis can also cause a decrease in growth hormone and testosterone production and may increase cortisol levels and the risk of insulin resistance, which can affect muscle protein synthesis and degradation pathways [21, 22]. In addition, sleep deprivation can cause disturbances in circadian rhythms, leading to imbalances in skeletal muscle metabolism [23].

A growing body of evidence supports the possibility that sleep disorders may be mediators of sarcopenia development. The concept of “possible sarcopenia” is relatively new, and its diagnostic criteria are less stringent than those for sarcopenia. Introducing “possible sarcopenia” to explore its association with sleep duration may help older people prevent and manage the risk of this condition in a more timely manner. However, the role of the association between sleep duration and possible sarcopenia is currently controversial and lacks support from a nationally representative sample, and validating this relationship could help establish good sleep models and provide effective intervention strategies beyond nutritional and exercise interventions. We hypothesize that sleep duration has a U-shaped relationship with possible sarcopenia, and we will verify this hypothesis in a retrospective longitudinal study using the CHARLS database.

## Methods

### Study population

The China Health and Retirement Longitudinal Study (CHARLS) aims to collect a set of high-quality microdata representing Chinese households and individuals aged 45 and above, to analyze the aging of China’s population, to promote interdisciplinary research on aging and to provide a more scientific basis for the formulation and improvement of relevant policies in China. The CHARLS national baseline survey was conducted in 2011, covering 150 county-level units, 450 village-level units, and 17,000 people in 10,000 households in 28 provinces, and these samples will be followed up every two to three years. The project uses a multistage sampling method proportional to population size (PPS). PPS sampling is also known as probability sampling proportional to size or probability proportional to volume sampling. The sampling principle of PPS is that the unequal probability of phases is exchanged for equal probabilities of the final and overall phases and covers basic personal information, household structure, health status, physical measurements, health service utilization and health insurance, basic community conditions, etc. To date, CHARLS has completed four waves of follow-up data collection, and detailed information about the CHARLS database has been published [24]. The project has also been approved by the Biomedical Ethics Committee of Peking University (IRB00001052-11015; IRB00001052-11014), and the data are available on the CHARLS website (http://charls.pku.edu.cn/index.htm).

**Fig. 1:**
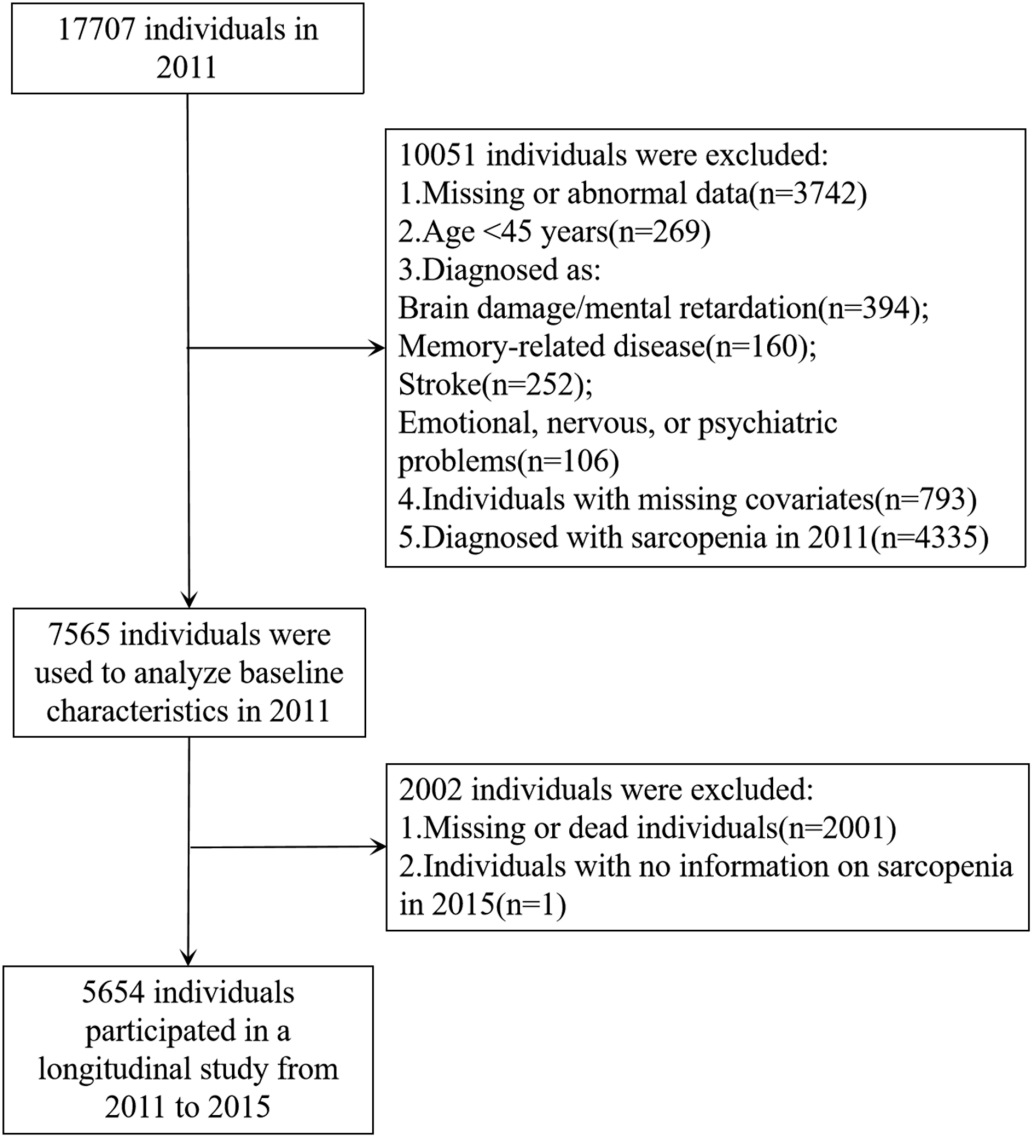
Flowchart of the sample selection process

This study proposed using the first wave of 2011 data and the third wave of 2015 data from the CHALRS database as survey respondents. A total of 17,707 individuals participated in the 2011 baseline survey, and we excluded 10,051 individuals because of (1) missing or abnormal data; (2) age < 45 years; or (3) having memory disorders (dementia, Parkinson’s, cerebral atrophy), brain damage/intellectual deficits, a history of stroke, or psychiatric/psychological problems; (4) missing covariates; (5) diagnosis of sarcopenia; a total of 7656 individuals participated in the first wave of the baseline analysis. In the longitudinal study, we excluded individuals who were lost to follow-up or died in wave 3 and lacked information on the diagnosis of possible sarcopenia; ultimately, we screened 5654 eligible participants. The details are shown in the images below (Fig. 1). It is important to note that some data such as height and weight have unit conversion problems and will be corrected after consulting the CHARLS forum. To ensure the authenticity and rigor of the data, data that cannot be corrected will be excluded, and these data will be classified as abnormal. Some abnormal grip strength values (such as 311 kg, 500 kg, etc.) were treated as missing values when comparing the other three groups and other years of measurement data.

### Assessment of possible sarcopenia

According to the relevant definition [7, 11], this study proposes to examine possible individuals with sarcopenia according to the AWGS 2019 [11] definition, i.e., meeting the criteria of low muscle strength (M < 28 kg, F < 18 kg) or poor physical performance (five sit-to-stand tests: ≥12 s). Participants were asked to squeeze a mechanical dynamometer (LeapfrogTM WL-1000, Nantong, China) as hard as possible [25]. Participants first stood, held the dynamometer at a right angle, and squeezed the handle for a few seconds. Each hand was tested twice, and the maximum grip force was taken for diagnosis. If, for some reason, it was not possible to measure one hand of the participant or if the grip strength value was abnormal, the maximum value was recorded for the other hand. Physical performance was measured by 5 repetitions of standing up from the chair with the respondent sitting with both arms crossed over the chest on a standard stool equipped with the program. The total time spent standing up straight and sitting down five times as fast as you can, without using your hands, was recorded [23].

### Sleep duration

The duration of nighttime sleep was mainly based on the subjective recollection of the participants, and the questionnaire was used to ask “In the past month, how many hours did you actually fall asleep each night on average? (probably shorter than the time you spent lying in bed)”, and then, based on the participants’ answers, the sleep duration was divided into three categories: short sleep duration (≤ 6 h), medium sleep duration (6–8 h), and long sleep duration (> 8 h) [26].

### Covariates

Based on a previous study [27, 28], we considered sociodemographic characteristics and health-related factors in our analysis. Sociodemographic characteristics included age, sex, area of residence (rural or urban), and educational level (< 9 and ≥ 9), and the participants were divided into two groups using junior high school graduation as the cutoff. Health-related factors included body mass index (BMI), ever/current smoking status, ever/current alcohol consumption, and five common comorbidities closely associated with sarcopenia (hypertension, hyperlipidemia, hyperglycemia, respiratory disease, and heart disease) [29, 30]. BMI was defined as weight (unit: kg) divided by the square of the height (unit: m). A BMI < 18.5 kg/m^2^ is considered underweight, 18.5–23.9 kg/m^2^ is considered normal weight, and ≥ 24 kg/m^2^ is considered overweight [31].

### Statistical analysis

All analyses will be performed using Stata 17.0 (https://www.stata.com/). This study analyzed wave 1 data from 2011 to determine the baseline characteristics of the different sleep time groups at that time point and to follow up with the same study population after screening for possible sarcopenia, using wave 3 data from 2015 for the same measures. ANOVA and chi-square tests were used to assess the underlying characteristics of the wave 1 data, with categorical variables expressed as frequencies and percentages and quantitative variables expressed as means and standard deviations. Univariate and multivariate logistic regressions were used to analyze the correlation between sleep duration and sarcopenia in the third wave of the data. The sleep duration was subjected to crude and adjusted models as independent variables, where Model 1 included age (≥ 65), sex (female), residence (rural), and education level (< 9 years); Model 2 included Model 1, ever/current smoking status, ever/current alcohol consumption, BMI, hypertension status, hyperlipidemia status, hyperglycemia status, respiratory disease status, and heart disease; and Sarcopenia (absent and probable) was used as the dependent variable. In addition, each subcomponent of the second wave data was submitted separately to the same model as the dependent variable. Following the same screening process, 7776 individuals with low grip strength and 5834 individuals with poor physical performance ultimately participated in the follow-up analysis. Odds ratios (ORs) and 95% confidence intervals (CIs) were calculated. The significance level of the statistical tests was set as *p* < 0.05 (two tailed).

## Results

**Fig. 2:**
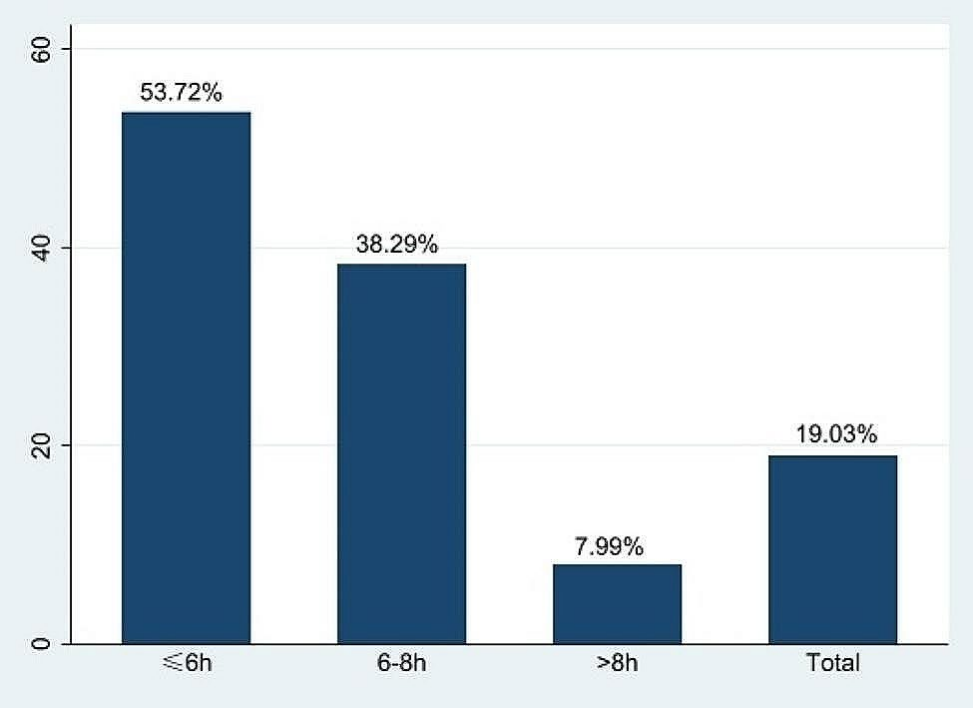
Prevalence of possible sarcopenia in different groups

In the first wave of data, a total of 7656 individuals were categorized as having nonsarcopenia. Among them, 3697 (48.29%) individuals had a short sleep duration, 3362 (43.91%) individuals had a medium sleep duration, and 597 (7.80%) individuals had a long sleep duration (Table 1). During follow-up, 2001 individuals were lost or died, as well as 1 individual lacking information for the diagnosis of sarcopenia, leaving 5654 individuals who participated in the first and third waves of the follow-up study. The prevalence of possible sarcopenia was 53.72% (578) in the short sleep duration group, 38.29% (412) in the normal sleep duration group, and 7.99% (86) in the long sleep duration group, for an overall sarcopenia incidence of 19.03% (1076). The prevalence of sarcopenia was relatively greater in the short sleep duration group. (Fig. 2)

**Table 1:** Baseline characteristics of individuals with nonsarcopenia in 2011

Variables	Total (*N* = 7656)	Short sleep (*N* = 3697)	Medium sleep (*N* = 3362)	Long sleep (*N* = 597)	*P*
**Age (years**)					
< 65	6266(81.84)	2947(47.03)	2836(45.26)	483(7.71)	0.000
≥ 65	1390(18.16)	750(53.96)	526(37.84)	114(8.20)	
**Gender**					
Male	3931(51.35)	1858(47.27)	1783(45.36)	290(7.38)	0.024
Female	3725(48.65)	1839(49.37)	1579(42.39)	307(8.24)	
**Residential area**					
Rural	735(9.60)	349(47.74)	355(48.02)	31(4.24)	0.000
Urban	6921(90.40)	3348(48.37)	3007(43.45)	566(8.18)	
**Education level**					
< 9	4749(62.03)	2395(50.43)	1935(40.75)	419(8.82)	0.000
≥ 9	2907(37.97)	1302(44.79)	1427(49.09)	178(6.12)	
**BMI(kg/m2)**					
< 18.5	402(0.55)	224(55.72)	146(36.32)	32(7.96)	0.000
18.5–23.9	4128(53.92)	2036(49.32)	1747(42.32)	345(8.36)	
≥ 24	3126(40.83)	1437(45.97)	1469(46.99)	220(7.04)	
**Ever/current smoke**					
Yes	3227(42.27)	1676(47.60)	1481(44.93)	248(7.47)	0.266
No	4429(57.85)	2161(48.79)	1912 (43.17)	356(8.04)	
**Ever/current alcohol**					
Yes	3405(44.47)	1676(4922)	1481(43.49)	248(7.28)	0.180
No	4251(55.53)	2021(4754)	1881(44.25)	349(8.21)	
**Comorbidities**					
High blood pressure	1525(19.92)	771(50.56)	633(41.51)	121(7.93)	0.101
High blood cholesterol	630(8.23)	314(49.84)	266(42.22)	50(7.94)	0.668
Hyperglycemia	390(5.09)	200(51.28)	161(41.28)	29(7.44)	0.477
Respiratory diseases	674(8.80)	375(55.64)	257(38.13)	42(6.23)	0.000
Heart disease	698(9.1)	374(53.58)	283(40.54)	41(5.87)	0.006

According to the univariate logistic regression analysis, compared with moderate and long sleep durations, short sleep durations were more strongly associated with the development of possible sarcopenia (*P* = 0.000) (Table 2, crude model). After adjustment for Model 1 and Model 2, the association between short sleep duration and possible sarcopenia was maintained (*P* = 0.029); however, no association was found between long sleep duration and possible sarcopenia. Furthermore, among the covariates, older age, rural residence, low education level, and underweight status were more likely to be associated with possible sarcopenia, and comorbidities in the sarcopenia group were significantly different (*P* < 0.05), as were hypertension, hyperlipidemia, and hyperglycemia.

**Table 2:** Association between sleep duration and possible sarcopenia based on logistic regression model analysis

Variables	(crude model)		(Model 1)		(Model 2)	
	OR (95% CI)	*P*	OR (95% CI)	*P*	OR (95% CI)	*P*
Sleep duration (h)						
≤ 6	1.35 (1.17–1.55)	0.000	1.20 (1.04–1.39)	0.010	1.18 (1.02–1.36)	0.029
6–8	Ref		Ref		Ref	
> 8	1.19 (0.92–1.54)	0.184	1.02 (0.78–1.33)	0.854	1.02 (0.78–1.34)	0.868
Age (≥ 65 years)			2.75 (2.39–3.18)	0.000	2.60 (2.25–3.01)	0.000
Gender (Female)			1.11 (0.96–1.28)	0.162	1.04 (0.84–1.28)	0.743
Residential area (rural)			1.23 (1.02–1.49)	0.026	1.25 (1.04–1.51)	0.020
Education (< 9 years)			1.80 (1.51–2.13)	0.000	1.78 (1.49–2.12)	0.000
Ever/current smoke (yes)					0.96 (0.79–1.17)	0.697
Ever/current alcohol (yes)					0.89 (0.76–1.05)	0.156
BMI (kg/m2)						
< 18.5					1.66 (1.27–2.18)	0.000
18.5–23.9					Ref	
≥ 24					0.96 (0.83–1.11)	0.587
Comorbidities						
High blood pressure					0.86 (0.72–1.02)	0.087
High blood cholesterol					1.13 (0.86–1.48)	0.374
Hyperglycemia					0.83 (0.60–1.13)	0.238
Respiratory diseases					0.73 (0.59–0.91)	0.005
Heart disease					0.71 (0.57–0.90)	0.004

Model 1: Age, gender, education, residence; Model 2: Model 1, Ever/current smoking status, Ever/current alcohol consumption status, BMI, hypertension, hyperlipidemia, hyperglycemia, respiratory diseases, heart disease

There may be changes in sleep duration at 4-year intervals, so we divided sleep duration into stable, slightly stable (spanning 1 group), and unstable (spanning 2 groups) according to whether there was an increase or decrease in sleep duration across the groups. Eventually, there were 3273, 2098 and 283 people in the three groups mentioned above, and after adjusting for confounders, neither the slightly stable group (OR: 1.10, 95% CI: 0.95–1.28, *P* = 0.194) nor the unstable group (OR: 1.25, 95% CI: 0.91–1.71, *P* = 0.165) was associated with sarcopenia; i.e., changes in sleep duration in this study did not affect the results. To further validate this conclusion, we reran the analysis after excluding the change group, and the results showed that short sleep duration was still strongly associated with sarcopenia even after adjusting for confounders (OR: 1.25, 95% CI: 1.02–1.52, *P* = 0.031). This result strongly supports the reliability of the findings of this study.

In the subgroup analysis, a total of 7776 participants were followed up in the grip strength group, and 5834 participants were followed up in the 5 chair stand test group. According to the crude model, short sleep duration was significantly associated with both low grip strength and poor physical performance (OR: 1.41, 95% CI: 1.21–1.65, *P* = 0.000 and OR: 1.31, 95% CI: 1.12–1.55, *P* = 0.001), while long sleep duration was only associated with low grip strength (OR: 1.41, 95% CI: 1.09–1.81, *P* = 0.009). However, in the logistic regression model, short sleep duration was only associated with low grip strength (*P* = 0.031) and not with poor physical performance. A long sleep duration was not associated with any of the components **(**Table 3). We also examined the effect of sleep duration on grip strength and performance in the 5 chair stand test cohort during follow-up, revealing that neither the slightly stable group (OR: 1.00, 95% CI: 0.86–1.17, *P* = 0.962; OR: 1.00, 95% CI: 0.84–1.17, *P* = 0.953) nor the unstable group (OR: 1.36, 95% CI: 0.99–1.86, *P* = 0.056; OR: 1.40, 95% CI: 1.00–1.98, *P* = 0.052) had a significant impact on the results.

**Table 3:** Association between sleep duration and parameters of Sarcopenia based on logistic regression analysis

Variables	Low grip strength (*N* = 7776)		Poor physical performance (*N* = 5834)	
Sleep duration (h)	OR (95%CI)	*P*	OR (95%CI)	*P*
≤ 6	1.20 (1.02–1.41)	0.031	1.16 (0.97–1.37)	0.097
6–8	Ref		Ref	
> 8	1.15 (0.88–1.50)	0.313	0.94 (0.68–1.30)	0.698

## Discussion

Our results suggest that short sleep duration is associated with possible sarcopenia in middle-aged and older adults. This relationship was maintained even after adjustment for sociodemographic characteristics and health-related factors. In addition, we found that short sleep duration was significantly associated with low grip strength.

The results of the present study suggest that short sleep duration is associated with sarcopenia in middle-aged and older adults, which is inconsistent with the findings of Korean and Japanese studies [28, 32], which concluded that long sleep duration was associated with sarcopenia, but the ages of the subjects in these two studies were not the same—20 years or older and 65 years or older—and the range of classifications for sleep duration differed. However, several previous cross-sectional studies have shown a U-shaped relationship between sleep duration and sarcopenia, i.e., either short or long sleep duration may be a risk factor for sarcopenia [33, 34]. Therefore, there is an inconsistent association between sleep duration and sarcopenia observed in the current study. Although the present study revealed a strong association between only short sleep duration and sarcopenia, a meta-analysis showed that the prevalence of sarcopenia was greater in those who were sleep deprived [20] and that older women with insomnia, low sleep quality and short sleep duration were more likely to be frail [35]. Moreover, there is evidence that short sleep duration is associated with impairment in multiple dimensions of sarcopenia, such as low muscle mass and muscle strength [18, 19, 36], and these findings were confirmed in our study.

One-third of one’s life is spent in sleep, and sleep is essential for maintaining a normal and healthy life. The changing lifestyles of the 21st century have made “sleep deprivation” an epidemic, with sleep deprivation weakening productivity levels, increasing the risk of death, and taking a heavy toll on modern economies [37]. The mechanisms associated with sleep deprivation and sarcopenia are not fully understood. Sleep deprivation may lead to circadian rhythm disorders [23], increased insulin resistance [38], increased plasma cortisol levels, decreased testosterone levels, and even reduced myogenic fibronectin synthesis, muscle weight and muscle fiber cross-sectional area [21, 22, 39], thereby affecting muscle protein homeostasis. Recent evidence suggests that shorter sleep also leads to increased gut proinflammatory bacteria [40] and induces chronic systemic hypoinflammation [41], followed by a decrease in insulin sensitivity via the gut-muscle axis, causing reduced skeletal muscle insulin sensitivity, activation of ubiquitin proteases and the autophagic lysosomal system [42], ultimately leading to decreased muscle mass and loss of muscle function. In addition, individuals who are short sleepers are unlikely to meet the normal activity recommendations [43]. Conversely, exercise may be a practical and effective means to mitigate sleep deprivation-induced mitochondrial dysfunction, insulin resistance, sarcoplasmic protein synthesis, and circadian rhythm changes [44]. Thus, exercise and sleep complement each other, and the combination of good sleep patterns and exercise may be a new and effective intervention strategy for improving sarcopenia.

The strengths of this study are the high quality of a longitudinal study based on data from a national Chinese sample to explain the causal relationship between sleep duration and sarcopenia and the representative sample of the CHARLS project using the PPS random sampling method. In addition, this study considered the effect of changes in sleep duration during the follow-up period on the results. This study also has several limitations. First, because the CHARLS database lacks a measure of muscle mass, this study utilizes a possible definition of sarcopenia from the 2019 AWGS, proposed for early identification of sarcopenia in primary care settings and communities, so the prevalence of sarcopenia in the sample may be greater than that identified for sarcopenia [45]. In addition, the CHARLS collects information on their spouse and other family members, and questionnaire burden may also affect the quality of responses. Second, we assessed sleep duration using a self-administered questionnaire from the CHARLS database. This has been reported to cause inconsistencies in objectively recorded sleep duration, leading to the possibility of false negatives and false positives [46]. Recall bias is also inevitable. Third, we did not consider the effect of sleep quality or daily variation in sleep duration on the results. Finally, we failed to address other covariates of sleep duration associated with sarcopenia, such as physical activity, medications, depression, and other disorders. We recommend using tools such as smart bracelets to measure objective sleep duration and DXA/BIA body composition analyzers to assess subjects with identified sarcopenia to reduce error.

## Conclusion

In conclusion, our longitudinal study concluded that short sleep duration was associated with possible sarcopenia in middle-aged and older adults, and more prospective studies with larger sample sizes are needed to confirm and further explore the underlying biological mechanisms involved. We recommend considering the effect of sleep parameters when performing sarcopenia prevention and treatment.

## Data Availability

The datasets generated and/or analyzed during the current study are available in the [CHARLS] repository. The URL is http://charls.pku.edu.cn/index.htm. The datasets used and/or analyzed during the current study are available from the corresponding author upon reasonable request.
